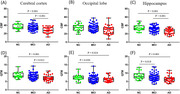# Quantitative transport mapping estimated blood flow velocity is more sensitive to the perfusion change in Alzheimer's disease than the conventional cerebral blood flow, estimated from multiple delay arterial spin labeling

**DOI:** 10.1002/alz.092455

**Published:** 2025-01-09

**Authors:** Liangdong Zhou, Yihao Guo, Yi Li, Yi Wang, Tao Liu, Huijuan Chen, Weiyuan Huang, Gloria Chiang, Mony J. de Leon, Feng Chen

**Affiliations:** ^1^ Weill Cornell Medicine, New York, NY USA; ^2^ Hainan General Hospital (Hainan Affiliated Hospital of Hainan Medical University), Haikou, Hainan China; ^3^ Weill Cornell Medicine, New York City, NY USA; ^4^ Brain Health Imaging Institute, Department of Radiology, Weill Cornell Medicine, New York City, NY USA

## Abstract

**Background:**

Quantitative transport mapping (QTM) has been developed for estimation of blood flow velocity with 4D dynamic tracer concentration data (Zhou et. al., Magn. Reson. Med. 2021;85: 2247–2262; Zhang et. al., Magnetic Resonance Imaging 2022; 86: 86–93). We investigated the QTM and CBF in human brain for Alzheimer's disease using multiple post‐labeling delay arterial spin labeling (mPLD‐ASL) MRI.

**Method:**

A total of 131 subjects (28 AD, 82 patients with mild cognitive impairment (MCI), and 21 normal controls (NC)) were prospectively recruited in this study. All participants underwent magnetic resonance imaging (MRI) examination and neuropsychological evaluation. CBF and QTM maps were computed from ASL with multiple PLD (Wang et al Neuroimage: Clinical 2013; 3:1‐7). Group differences of regional CBF and QTM analysis were performed and compared. The associations between perfusion and cognition were assessed.

**Result:**

Regional analysis showed decreased QTM value in occipital lobe, and hippocampus in MCI compared with NC as shown in Figure 1 (D)‐(F). CBF only decreases in AD patients compared to NC in cerebral cortex and hippocampus and was not able to distinguish MCI and NC as shown in Figure 1 (A)‐(C). CBF and QTM in hippocampus were both positively correlated with cognition, including global cognition, memory, executive function, and language function.

**Conclusion:**

This study demonstrated a reduced QTM of occipital lobe and hippocampus in MCI compared with NC, suggesting QTM as a potential early biomarker for AD. QTM in the hippocampus are correlated with cognitive deterioration. These findings contribute to a clear comprehension of perfusion change, glymphatic clearance deficit and cognitive decline in AD. QTM performs superior to CBF in distinguishing MCI and NC could be due to its accurate biophysical nature where arterial input function (AIF) is not required, and local mass conservation is satisfied. Global AIF used in CBF estimation could be not sufficiently sensitive to the physiological change at the early stage of AD.